# Editorial: Cytoskeleton in the focus of anti-cancer therapy: In a search of novel biomarkers and combinatorial therapy approaches

**DOI:** 10.3389/fphar.2022.1069821

**Published:** 2022-11-24

**Authors:** I. Vorobjev

**Affiliations:** ^1^ Department of Biology, School of Sciences and Humanities, Nazarbayev University, Nur-Sultan, Kazakhstan; ^2^ National Laboratory Astana, Nazarbayev University, Nur-Sultan, Kazakhstan

**Keywords:** cytoskeleton, cancer markers, anti-cancer therapy, microtubules (MT), actin

The development of targeted chemotherapy demands detailed molecular analysis of cancers to generate precision chemotherapy programs. While standard differential cancer diagnostic is based on the morphological features of cancer tissue with the inclusion of some molecular characteristics, a more comprehensive characterization of cancer cells focused on the upregulation or downregulation of specific proteins for highly efficient therapy is needed. In response to this demand, we collected this Special Issue.

Analyzing actin cytoskeleton in the current volume, Dugina et al. discuss the differential expression of actin isoforms in cancer cells with emphasis on the correlation of expression profile with cancer cell motility. Epithelial cells express two major actin isoforms—β-actin and γ-actin with clear spatial separation between them. A growing body of evidence shows that elevated level of γ-actin directly correlates with enhanced cell motility and cancer progression. Of special interest might be the down-regulation of β-actin in tumor tissues. This raises a question about possible alternative regulation of actin isoforms during cancer development. Search for the factor(s) regulating actin isoform expression is of great interest and requires further investigation.

In cancer cells, some signaling pathways may be altered or completely lost. This feature often results in the uncontrolled growth of cancer cells. Interestingly, alteration or loss of normal regulatory pathways could be used for anti-cancer therapy. One of the examples is the loss of substrate rigidity sensing discussed by Tijore et al. in the current volume. Several proteins, including tropomyosin 2.1, filamin A, and myosin IIA involved in rigidity sensing, are downregulated during cancer development. This makes cells sensitive to mechanoptosis—a physical treatment that selectively kills cancer cells while normal counterparts easily resist it.

The ability of cancer cells to switch from mesenchymal to amoeboid types of motility allows them to obtain the enhanced capacity to form metastases after several standard anti-cancer therapy treatments like radiotherapy, the use of angiogenesis inhibitors, etc. It also happens as a side effect of well-known microtubule targeting agents (taxanes, Vinca alkaloids). Alexandrova and Lomakina, in the current volume, focus on the migratory plasticity of cancer cells and discuss the usefulness of specific proteins associated with the actin cytoskeleton, namely filamin A, fascin 1, podoplanin (PDPN), and alpha-actinins 1 and 4 as markers predicting migratory plasticity of cancer cells and possible outcome of the standard anti-cancer treatments.

Among different proteins associated with the actin cytoskeleton, Arp2/3 complex is of special interest. It is one of the master complexes driving actin polymerization at the leading edge and stimulating the formation of the cell’s lamellipodia. Since components of Arp2/3 complex are overexpressed in a variety of cancers (colorectal, liver, lung, and breast cancers) and their overexpression always correlates with poor prognosis, inhibitors of Arp2/3 components might be beneficial for targeted treatments of advanced cancers. Fokin et al. in the current volume describe the synthesis of novel potent Arp2/3 inhibitors. Remarkably, these new low molecular weight analogs of Arp 2/3 inhibitor CK-666 show a higher effect *in vivo* (on cultured cells and in sea urchin embryo development) while having slightly lower chemical affinity to the target molecule compared to the CK-666 itself.

Microtubules are another cytoskeletal target for anti-cancer programs. Wattanathamsan and Pongrakhananon considered the role of microtubule associated proteins (MAPs) in the microtubule dynamic and its relation to cancer prognosis. Increased dynamics of microtubules result from the up-regulation of different proteins interacting with microtubules, i.e., CAMSAP2, katanin, stathmin, and KIF14. Importantly these upregulations of the MAPs that are ubiquitously expressed in different tissues are often associated with cancer metastasis (Wang et al., 2020; Zhang et al., 2020). Authors conclude that elevated microtubule dynamics in cancer cells usually (if not always) correlate with poor prognosis. This review highlights MAPs as a novel group of prognostic markers and potential objects for targeted chemotherapy.

Combinatorial treatment using microtubule targeting agents (MTA) together with other antineoplastic agents has become a popular approach (Liang et al., 2022). However, the question of how to create the most effective combination of these drugs remains open. One opportunity is the combination of MTAs with inhibitors of anti-apoptotic proteins (Whitaker and Placzek, 2019). Suleimenov et al. in the current volume demonstrate that during mitotic arrest, cells become primed to apoptosis, and nanomolar doses of the inhibitors of anti-apoptotic protein bcl-xL, but not of other bcl-2 proteins induce death in mitosis of cells resistant against mitotic arrest. This effect is observed when the duration of mitotic arrest exceeds 4 h and cells undergoing mitotic slippage within several hours become insensitive to the bcl-xL inhibitors.

Another approach to combinatorial treatment is described in the current volume by Lafanechère et al., who discusses the use of combinations of drugs with different binding sites to the tubulin molecule. Recent data shows a synergistic effect when using novel microtubule destabilizing carbazole, Carba 1, together with microtubule stabilizer paclitaxel (PTX) (Peronne et al., 2020). This synergy allows the reduction of PTX dose, thus minimizing its side effects, and whether carbazole will be well tolerated *in vivo* might become a highly prospective therapy program.

Summing up, I expect that this research topic will give useful insights into our understanding of the cytoskeleton as an object for precise differential diagnostics of tumors and will be helpful for the development of novel targeted therapy programs.
